# Prenatal Diagnosis of Isolated Congenitally Corrected Transposition of the Great Arteries

**DOI:** 10.7759/cureus.103097

**Published:** 2026-02-06

**Authors:** Aditi R Chavhan, Jitendra Sahu, Kajal Mitra, Suresh Phatak, Prashant Onkar

**Affiliations:** 1 Department of Radiodiagnosis and Imaging, NKP Salve Institute of Medical Sciences and Research Centre, Nagpur, IND

**Keywords:** congenitally corrected transposition of the great arteries, discordance, echocardiography, fetal, prenatal diagnosis

## Abstract

Congenitally corrected transposition of the great arteries (ccTGA) is an uncommon congenital heart defect characterized by atrioventricular and ventriculo-arterial discordance resulting from abnormal leftward looping of the primitive cardiac tube. Although this double discordance produces a physiologically corrected circulation, the morphologic right ventricle functions as the systemic ventricle, and the tricuspid valve serves as the systemic atrioventricular valve, predisposing affected individuals to late complications. Prenatal diagnosis of ccTGA is often challenging, particularly in isolated cases without associated cardiac anomalies, as the four-chamber view may appear deceptively normal on routine obstetric ultrasound. In current prenatal cohorts reported in the literature, isolated ccTGA without additional structural cardiac anomalies is uncommon. In several series of prenatally diagnosed ccTGA, only about 13-15% of cases were isolated when postnatal findings were also considered.

We report a case of isolated ccTGA diagnosed at 24 weeks and 4 days of gestation through detailed fetal echocardiographic evaluation and confirmed postnatally. Compared with the routine anomaly scan, which assesses only basic cardiac views (situs, four-chamber, and outflow tracts), a dedicated fetal echocardiography was performed. A sequential segmental approach was used, including confirmation of situs, detailed four-chamber morphology, atrioventricular and ventriculo-arterial connections, outflow tract and three-vessel views, aortic and ductal arches, and cranio-caudal sweeps from abdomen to mediastinum were used to track chamber continuity, great artery relationships, and color Doppler evaluation of flow across the valves and great vessels. Systematic assessment demonstrated ventricular inversion, with the systemic ventricle showing coarse trabeculations and a moderator band, along with apical displacement of the systemic atrioventricular valve consistent with tricuspid morphology. Atrioventricular discordance was clearly identified, and the great arteries were observed to run in a parallel orientation with abnormal ventriculo-arterial connections, key echocardiographic features that enabled accurate prenatal diagnosis.

Importantly, no additional intracardiac anomalies were detected. The neonate remained clinically stable after birth, with a normal sinus rhythm and no evidence of immediate hemodynamic compromise. This case highlights the importance of a structured fetal cardiac evaluation extending beyond the standard four-chamber view. Careful attention to ventricular morphology, atrioventricular valve offsetting, and the spatial relationship of the great arteries is essential for identifying isolated ccTGA.

Early prenatal diagnosis facilitates appropriate parental counseling, optimized perinatal planning, and structured long-term follow-up. Children with congenitally corrected transposition of the great arteries require lifelong surveillance because of the risk of progressive systemic right ventricular dysfunction, tricuspid valve regurgitation, arrhythmias, and conduction abnormalities, and early recognition allows timely intervention and improved functional outcomes into adolescence and adulthood.

## Introduction

Isolated congenitally corrected transposition of the great arteries is uncommon in prenatal cohorts. Current series report that only about 13-22% of prenatally diagnosed ccTGA cases are isolated without major associated cardiac anomalies [1]. Isolated ccTGA refers to atrioventricular and ventriculo-arterial discordance without other structural cardiac defects, whereas most ccTGA cases have associated anomalies such as ventricular septal defects, pulmonary outflow tract obstruction, and tricuspid valve abnormalities [2].

The anomaly develops when the embryonic heart tube loops abnormally to the left (L-looping), resulting in inversion of the ventricles and the characteristic mismatch between both atrioventricular and ventriculo-arterial connections [3,4]. As a result, the structurally right-sided ventricle is positioned on the left and functions as the systemic ventricle, exposing it to long-term high-pressure load, increasing the likelihood of ventricular dilation, hypertrophy, and eventual decline in systolic function [5].

In this configuration, the tricuspid valve becomes the systemic atrioventricular valve, making it vulnerable to regurgitation, which is aggravated by intrinsic structural variations, displaced positioning, or associated abnormalities resembling Ebstein malformation [6,7]. The atypical course of the conduction pathways further increases the likelihood of developing progressive rhythm disturbances, particularly complete heart block [8].

Prenatal recognition of this condition is often difficult because the standard four-chamber view may appear normal. Therefore, meticulous assessment of ventricular structure, the alignment of the atrioventricular valves, and the course and relative positioning of the great arteries becomes essential when attempting to identify isolated ccTGA [9].

Prenatal recognition of isolated ccTGA helps tailor perinatal care by prompting delivery planning at a facility with neonatal cardiology expertise and anticipating the need for early postnatal evaluation and monitoring, even in the absence of major associated defects. Serial fetal echocardiography can detect evolving hemodynamic changes and guide counseling about timing and mode of delivery, as well as early postnatal management and follow-up strategies to monitor for rhythm abnormalities or ventricular function changes that may influence outcomes. These prenatal insights into cardiac anatomy and progression have been shown to inform counseling and perinatal management planning in ccTGA [10].

## Case presentation

A 24-year-old healthy primigravida was referred at 24 weeks and 4 days of gestation for suspected transposition of the great arteries following a routine obstetric ultrasound. No congenital heart disease was reported in the family, and the mother had no significant medical conditions during pregnancy, including gestational diabetes or any exposure to known teratogens.

Prenatal echocardiography

A targeted fetal echocardiogram was performed using a high-end ultrasound system capable of 2D imaging, color Doppler, spectral (pulse-wave) Doppler, and M-mode. A transducer with appropriate frequencies (typically up to 3.5-7 MHz) and settings optimized for a high frame rate was used to visualize the fetal heart and maximize image quality.

Systematic sequential segmental assessment started with abdominal and atrial situs, cardiac position, and axis. Standard structural views included four-chamber view, left and right ventricular outflow tract views, three-vessel view, and three-vessel-trachea view to evaluate the great arteries. Additional imaging included aortic arch and ductal arch views, short-axis ventricular sweeps, and bicaval and long-axis planes. Gray-scale imaging was combined with color Doppler to assess flow across valves, venous connections, and shunts. Color Doppler was used to detect flow direction and disturbances through atrioventricular and semilunar valves, pulmonary veins, and the ductus arteriosus. Fetal cardiac rhythm was evaluated by calculating cycle lengths with Doppler across atrial and ventricular structures to confirm normal AV conduction and exclude bradyarrhythmias or heart block prenatally.

**Figure 1:**
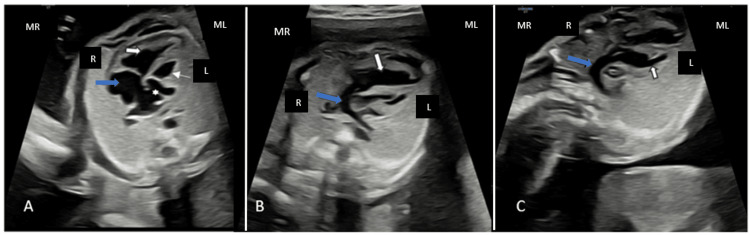
Four-chamber view A. Inversion of the ventricles, with the morphological right ventricle (RV) (thin arrow) positioned on the left; the morphological left ventricle (LV) positioned on the right (white arrow); the left atrium (star) connected to the morphological right ventricle (RV); the right atrium (blue arrow) connected to the morphological left ventricle (LV) B. Pulmonary artery (blue arrow) originating from the morphological left ventricle (LV) (white arrow) C. Aorta (blue arrow) originating from the morphological right ventricle (RV) (white arrow) Maternal right (MR), Maternal left (ML), Fetal right (R), Fetal left (L)

In the four-chamber view, an inversion of the ventricles was observed, with the morphological right ventricle situated on the left side and the morphological left ventricle positioned on the right. The left atrium was found to drain into the morphological right ventricle, while the right atrium connected to the morphological left ventricle (Figure 1A). Additionally, the pulmonary artery was seen arising from the morphological left ventricle (Figure 1B), whereas the aorta originated from the morphological right ventricle (Figure 1C).

**Figure 2:**
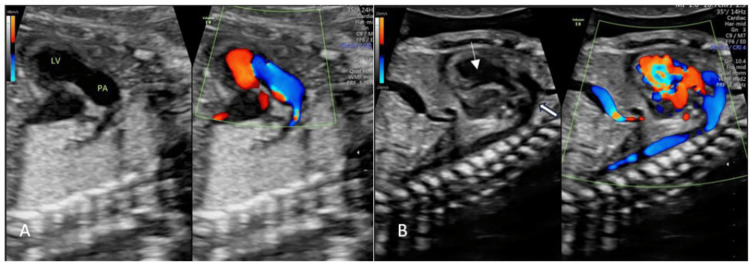
Color Doppler imaging A. The pulmonary artery originating from the morphological left ventricle (LV); B. The aorta (white arrow) originating from the morphological right ventricle (RV) (thin arrow)

Colour Doppler imaging demonstrated the pulmonary artery arising from the morphological left ventricle (Figure 2A). The aorta was visualized originating from the morphological right ventricle (Figure 2B).

No ventricular septal defects, outflow obstruction, or systemic AV valve abnormalities were observed.

Birth and postnatal findings

A female newborn was delivered by cesarean section at 38 weeks’ gestation, with Apgar scores of 9 and 10 and a weight of 2898 g. Clinically, the baby was asymptomatic, so no intervention was planned by the clinician. The newborn remained clinically stable and was discharged on day four.

**Figure 3:**
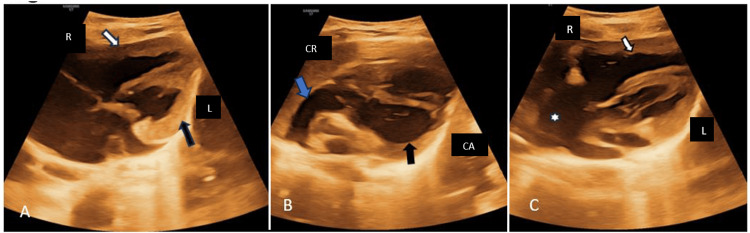
Postnatal echocardiography A. The morphological right ventricle (RV) (black arrow) is positioned on the left, and the morphological left ventricle (LV) (white arrow) is positioned on the right, indicating ventricular inversion. B. The aorta (Blue arrow) originates from the morphological right ventricle (RV) (arrow). C. The pulmonary artery (star) originates from the morphological left ventricle (LV) (arrow). Right (R), Left (L), Cranial (CR), Caudal (CA)

Postnatal echocardiography was performed after six months, which confirmed AV and VA discordance (Figure 3A). The aorta was observed arising from the morphological right ventricle (Figure 3B), and the pulmonary artery was seen originating from the morphological left ventricle (Figure 3C), with no additional anomalies.

At the six-month follow-up visit, the child showed normal growth and had no reported symptoms. A follow-up 2D echocardiogram was suggested.

## Discussion

ccTGA is an infrequently encountered congenital heart defect caused by an atypical leftward (L-loop) turn of the embryonic cardiac tube. This developmental deviation reverses the ventricular positions and gives rise to the combined AV and VA discordance typical of the condition [3]. Because the physiologic circulation remains preserved, isolated ccTGA often demonstrates subtle prenatal imaging clues and may be difficult to diagnose on routine screening, particularly when unaccompanied by additional structural defects.

Prenatal echocardiography is central to identifying ccTGA, beginning with recognition of ventricular morphology. The structurally right-sided ventricle is identified by a moderator band and coarse trabeculations, while the structurally left-sided ventricle has smoother myocardial walls. Apical displacement of the tricuspid valve is another helpful marker for identifying the systemic ventricle in fetuses with this condition [9]. Evidence of AV and VA discordance is noted when the left atrium connects to the structurally right-sided ventricle and the right atrium to the structurally left ventricle, which assists in confirming ventricular inversion during fetal evaluation. Examination of the outflow tracts often shows the great vessels running in parallel instead of their usual crossed relationship, with the aorta frequently positioned anteriorly and to the left--findings characteristic of ccTGA and reflective of conotruncal malalignment within the transposition spectrum [5]. On the three-vessel and trachea view, the expected “V-shaped” arrangement is usually absent; in some fetuses, a more linear “L-shaped” configuration may be seen, pointing toward a form of transposition [7].

All the principal echocardiographic markers described for congenitally corrected transposition of the great arteries were identified in this fetus, including ventricular inversion, atrioventricular and ventriculo-arterial discordance, apical displacement of the systemic tricuspid valve, and parallel great arteries on outflow tract views. No septal defects, outflow obstruction, or other associated abnormalities were seen, consistent with an isolated form of ccTGA.

The prenatal features observed in our case are consistent with recent reports in the literature. Hematian et al. described a case of isolated ccTGA diagnosed at 18 weeks’ gestation during routine screening, where careful assessment of ventricular morphology and connections allowed early identification of atrioventricular and ventriculo-arterial discordance despite the absence of other major cardiac anomalies [11].

A critical aspect of prenatal cardiac assessment in suspected ccTGA is distinguishing it from other congenital heart defects that can present with similar basic views, particularly d-transposition of the great arteries (d-TGA) and isolated ventricular inversion without arterial discordance. In ccTGA, systematic segmental analysis reveals double discordance, both atrioventricular and ventriculo-arterial, with the morphological right ventricle supporting systemic circulation and the morphological left ventricle supporting the pulmonary circuit. In contrast, d-TGA demonstrates concordant atrioventricular connections with only ventriculo-arterial discordance, typically seen as a posteriorly arising aorta from the left ventricle and an anterior pulmonary artery from the right ventricle. Isolated ventricular inversion shows discordant atrioventricular connections but normal ventriculo-arterial connections, lacking the great artery transposition characteristic of ccTGA. Careful evaluation of ventricular morphology (including trabecular pattern and moderator band), atrioventricular valve relationships, and outflow tract origins on multiple planes helps differentiate these entities prenatally, avoiding misclassification [5].

Although the circulation remains “physiologically corrected,” ccTGA carries important long-term risks because the structurally right-sided ventricle functions as the systemic ventricle. With time, this ventricle can begin to fail, and patients may develop worsening of tricuspid valve leakage or even complete heart block, complications that are particularly common as these children grow older [12].

Early gestation diagnosis of ccTGA is difficult because the small fetal heart and evolving anatomy can obscure ventricular morphology and outflow relationships, leading to potential misclassification; careful segmental analysis and follow-up imaging improve accuracy [13].

Isolated ccTGA generally does not require immediate neonatal surgical intervention; however, accurate antenatal diagnosis is essential for delivery planning, parental counseling, and establishing lifelong cardiology follow-up to monitor systemic RV performance and conduction disturbances.

ccTGA is frequently associated with additional cardiac defects that influence prognosis and management. Ventricular septal defects, pulmonary outflow tract obstruction, and systemic atrioventricular (tricuspid) valve abnormalities are the most common associations and may necessitate early intervention or surgery, while conduction disturbances such as atrioventricular block can affect long-term outcomes. Extracardiac anomalies are less frequent but should be actively screened for, as their presence may alter counseling and perinatal planning [5].

Fetal MRI serves as a useful adjunct to ultrasound-based cardiac evaluation in the antenatal evaluation of ccTGA, particularly when acoustic windows are limited by maternal habitus, oligohydramnios, or fetal position [14]. MRI provides high-contrast, multiplanar visualization that helps confirm ventricular morphology, delineate AV and VA relationships, and better demonstrate the parallel great arteries with an anterior, left-sided aorta, characteristic of ccTGA [15]. It is not a replacement for fetal echo; however, it provides valuable supplementary anatomical information, especially in complicated cases or when ultrasound findings are uncertain.

Fetal MRI should be recommended when ultrasound findings are equivocal, limited by maternal habitus or fetal position, or when extracardiac anomalies are suspected. In cases of isolated ccTGA with clear and comprehensive echocardiographic views, normal growth parameters, and no suspicion of extracardiac anomalies, fetal MRI remains optional [16].

## Conclusions

This case highlights the importance of meticulous fetal echocardiographic assessment in facilitating accurate mid-trimester (24 + 4 weeks’ gestation) prenatal diagnosis of isolated congenitally corrected transposition of the great arteries (ccTGA). The recognition of characteristic features, such as ventricular inversion, abnormal tricuspid valve positioning, and the parallel orientation of the great arteries, allowed for timely and accurate identification of the anomaly. The prenatal identification of ccTGA enabled planned delivery at a center with pediatric cardiology support and facilitated a structured postnatal follow-up strategy, including early echocardiographic confirmation, periodic monitoring of systemic right ventricular function and tricuspid valve competence, and routine rhythm surveillance with ECG to detect conduction abnormalities. Despite isolated anatomy, counseling should address the potential for long-term complications, including progressive systemic right ventricular dysfunction, tricuspid valve regurgitation, and conduction abnormalities, as these may develop later in childhood or adulthood and necessitate lifelong cardiology follow-up.
